# A Study on the Effect of Indirect Nitrate Supply on the Nitrogen Fixation Capacity of Soybean Nodules

**DOI:** 10.3390/plants13243571

**Published:** 2024-12-21

**Authors:** Sha Li, Huidi Hu, Baiyang Yu, Liwen Han, Wei Li, Zhilei Liu, Xuesheng Liu, Xiaochen Lyu, Zhenping Gong, Chunmei Ma

**Affiliations:** 1 College of Resources and Environment, Northeast Agricultural University, Harbin 150030, China; risa_shasha@163.com (S.L.); weili@neau.ed.cn (W.L.); hlliuzhilei@126.com (Z.L.); liuxuesheng0101@163.com (X.L.); 2 College of Agriculture, Northeast Agricultural University, Harbin 150030, China; 15214598488@163.com (H.H.); ybyyby0611@163.com (B.Y.); 15776616685@163.com (L.H.); xiaochenlyu@163.com (X.L.); gzpyx2004@163.com (Z.G.)

**Keywords:** soybean, nodule, nitrogenase activity, infected cells, uninfected cells, starch, sucrose

## Abstract

In this study, dual-root soybean (*Glycine max* L. Merr.) plants, with one side nodulated and the other nonnodulated, were used as experimental materials. The nonnodulated lateral roots were treated with excessive nitrate (200 mg·L^−1^ nitrogen) for three days, followed by a three-day nitrate withdrawal, and then subjected to excessive nitrate again for another three days. Meanwhile, the nodulated side was continuously supplied with nitrogen-free nutrient solution. We measured the nitrogenase activity, nodule quantity, and concentrations of sucrose, starch, and soluble sugars, along with the microstructure of the nodules. By analyzing these data, we aim to provide theoretical insights into how indirect nitrate supply affects the nitrogen fixation capacity of nodules. The results demonstrated that indirect supply of nitrate to the soybean nodules reduced the nodule nitrogen fixation ability, which was manifested in the decrease in nodule dry weight, nodule number, and nitrogenase activity. The reason was found to be related to the decrease in carbon sources (sucrose, starch, and soluble sugar) allocated to the nodules. Further observation of the internal structure of the nodules found that the number of infected cells in the nodules decreased with the addition of nitrate, and increased with its withdrawal. However, the addition and withdrawal of nitrate did not change the effect of nitrate on the structure of infected cells around the nodules after the first addition of nitrate. These may be one of the important reasons why nitrate indirectly affects the activity of nitrogenase in nodules.

## 1. Introduction

Nitrogen (N) is a key factor in plant growth and productivity. The symbiotic relationship between legumes and bacteroids allows legumes to utilize atmospheric N_2_ for their own N needs [1]. Although legumes have the ability to form N_2_-fixing symbioses, several factors can impede nodulation. As a result, inorganic N sources such as nitrate (NO_3_^−^) and ammonium (NH_4_^+^) are as vital to legumes as they are to non-legumes [2].

Excessive nitrate can significantly inhibit N fixation in legume nodules [3,4,5]. Supplying nitrate to bean (*Phaseolus vulgaris*) or soybean (*Glycine max* L. Merr.) plants for 1 day significantly decreased their nodule nitrogenase activity [6,7]. When nitrate was applied to soybean plants for one week, nodule growth was inhibited. However, when nitrate was removed, nodule growth resumed [8]. Using dual-root soybeans as experimental materials, one root system was supplied with nitrate, while the other received a N-free nutrient solution. The nitrogenase activity of nodules on the N-supplied side was significantly lower than that on the N-free side. It is proposed that nitrate inhibits nodule nitrogenase activity by directly affecting the nodules, suppressing their growth, which subsequently leads to a decrease in the overall nitrogenase activity of the soybean plant [9]. Importantly, this inhibitory effect is recoverable. When nitrate was withdrawn after its application, the nitrogenase activity in the nodules gradually recovered [10]. Nitrate application also influenced the distribution of carbohydrates across various plant organs. The inhibition of nodule nitrogenase activity by nitrate was found to be associated with a reduction in the supply of photosynthetic assimilates to the nodules [3,11]. Specifically, nitrate application promoted the transport of carbohydrates to the roots, stimulating root growth to enhance nitrate absorption. This, in turn, reduced the allocation of carbohydrates to the nodules, decreasing their carbohydrate utilization and further suppressing nitrogenase activity [8,12]. However, not all studies agree with this interpretation. James and Minchin reported that when 10 mM nitrate was supplied to soybeans, the levels of sucrose and hexose in the nodules remained unchanged, and the peak value of nitrate absorption did not coincide with the decline in nitrogenase activity. They concluded that the distribution of carbohydrates in the nodules was not related to the initial decline in nitrogenase activity in the nodules [13]. Therefore, the relationship between nitrate, carbohydrate distribution, and nodule N fixation capacity remains a topic of interest for many researchers.

When nitrate reduces the carbon supply to nodules, structural changes in the nodules can occur. Photosynthate availability affects the maturation, senescence, and distribution of bacteroids within alfalfa (*Medicago sativa* L. cv. Buffalo) nodules [14]. In soybeans, dark stress can decrease sucrose content in nodules by up to 84%, and carbohydrate deprivation leads to the rearrangement of infected cells in nodules [15]. For instance, the volume of uninfected cells in the central region of soybean nodules without nitrate supply accounts for 21% of the total volume, whereas in plants exposed to nitrate, uninfected cells make up 31% of the central region volume [16].

In a previous study, we used dual-root soybean systems with nodules on only one side, and applied nitrate to the nonnodulated side to provide an indirect nitrate supply to the nodules, thereby avoiding the direct toxic effects of nitrate contact with nodules. This resulted in decreased nitrogenase activity and structural changes in the nodules [4,10]. However, it remains unclear how nodule structure changes after nitrate withdrawal. The focus of this study is to determine whether structural changes in nodules after nitrate supply and withdrawal are linked to changes in carbohydrate supply and, ultimately, to the impact of nitrate on nodule N_2_ fixation capacity.

## 2. Results

### 2.1. Soybean Nodule N Fixation Ability Affected by Nitrate

The addition and subsequent withdrawal of nitrate had a notable effect on nodule growth (Table 1). The number of nodules in the nonnodulated side supplied with nitrate for 3 days (N_HHH_ treatment phase I) was 9.7% lower than that in the control (N_LLL_ treatment), and there was no significant difference between the two treatments. When the nitrate supply days increased to 6 days (N_HHH_ treatment phase II) and 9 days (N_HHH_ treatment phase III), the number of nodules in the N_HHH_ treatment was significantly lower than that in the N_LLL_ treatment in phase II and phase III by 18.9% and 23.2%, respectively. These indicated that a high concentration of nitrate supplied to the nonnodulated side significantly inhibited nodule formation on the nodulated side. Supplying nitrate for 3 days then withdrawal for 3 days (N_HLL_ treatment phase II) was not significantly different from the N_HHH_ and N_LLL_ treatments in phase II, but the nodule number was 9.7% higher than the N_HHH_ treatment in phase II. When the nitrate withdrawal days increased to 6 days (N_HLL_ treatment phase III), the number of nodules was 12.1% lower than that of the N_LLL_ treatment and 14.2% higher than that of the N_HHH_ treatment in phase III, and there was a significant difference between the two treatments. These findings suggest that the number of nodules gradually increased as the number of days without nitrate on the nonnodulated side increased after nitrate withdrawal. After nitrate supply/withdrawal/resupply (N_HLH_ treatment in phase III), there was no significant difference between the N_HLH_ treatment and N_HLL_ treatment, but the number of nodules was 2.6% higher than that of the N_HHH_ treatment and 9.6% lower than that of the N_HLL_ treatment. This pattern indicates that the number of nodules on the nodulated side decreased, increased, and then decreased again in response to the nitrate supply/withdrawal/resupply pattern on the nonnodulated side. The dry weight of nodules followed a similar trend to the nodule count, with nodule dry weight decreasing when nitrate was supplied to the nonnodulated side and increasing upon nitrate withdrawal. These results demonstrate that the indirect supply of nitrate to soybean nodules inhibited their growth. Upon nitrate withdrawal, nodule growth gradually recovered, with the rate of recovery increasing as the duration of nitrate withdrawal extended.

The indirect supply of nitrate to soybean roots significantly impacted acetylene reduction activity (ARA), measured in μmol of ethylene produced per plant per hour, as well as the specific nitrogenase activity (SNA), measured per gram of dry nodule weight per hour (Table 2). During the three phases of continuous nitrate supply to the nonnodulated side (N_HHH_ treatment), ARA decreased by 42%, 47%, and 56% compared to the control (N_LLL_ treatment). When nitrate was supplied in phase I and then withdrawn in phases II and III (N_HLL_ treatment), ARA was reduced by 38% and 5%, respectively, compared to the control, indicating that after nitrate withdrawal, ARA gradually recovered and approached control levels by phase III. In the N_HLH_ treatment, where nitrate was supplied to the nonnodulated side, withdrawn, and then resupplied, ARA rapidly decreased again. The trends in SNA mirrored those of ARA across all phases. These results indicate that the indirect supply of nitrate to the nodulated side can modulate the N fixation capacity of soybean nodules. Although short-term nitrate exposure reduces nodule N fixation capacity, it can recover once nitrate is withdrawn.

### 2.2. Soybean Nodule Carbohydrate Concentration Affected by Nitrate

The concentrations of sucrose, starch, and soluble sugars (Table 3) in soybean nodules were significantly influenced by nitrate supply. During the first three days of nitrate supply to the nonnodulated side (N_HHH_ treatment, phase I), there was no significant change in sucrose concentration compared to the control (N_LLL_ treatment). However, as the nitrate supply period extended (6–9 days) (N_HHH_ treatment, phases II and III), the sucrose concentration in the nodules decreased significantly compared to the control. As nitrate was withdrawn (N_HLL_ treatment, phases II and III), the sucrose concentration in the nodules gradually increased. The trends for starch and soluble sugar concentrations followed a similar pattern to that of sucrose. These findings suggest that the indirect supply of nitrate to the nodules negatively regulated the concentrations of sucrose, starch, and soluble sugars, with these concentrations decreasing as nitrate supply increased and recovering as nitrate was withdrawn.

### 2.3. Soybean Nodule Microstructure Affected by Nitrate

**Figure 1:**
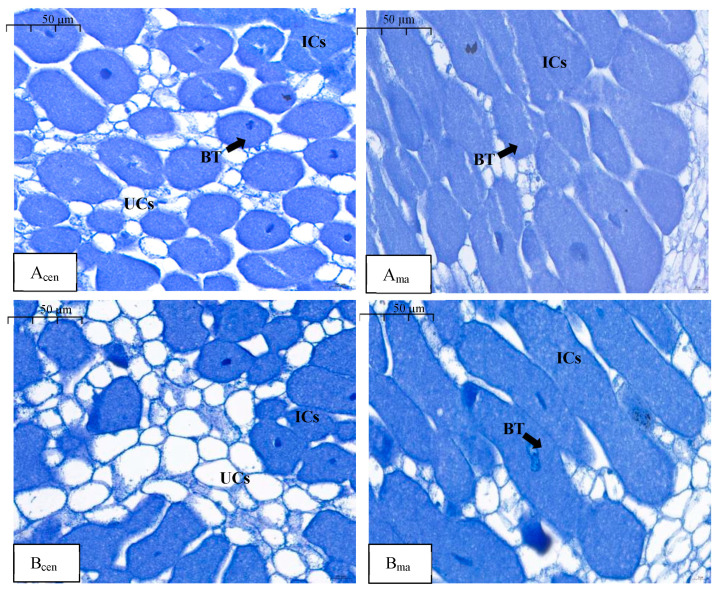
Microstructure of nodules in the N_LLL_ treatment (A_cen_ and A_mag_) and N_HHH_ treatment (B_cen_ and B_mag_) in phase I. BT: rhizobium, UCs: uninfected cells, ICs: infected cells; scale bar = 50 µm.

At the end of phase I, in the N_LLL_ treatment, infected cells (ICs) and uninfected cells (UCs) were evenly distributed in the center of the nodule. Using Photoshop and scale bars, we calculated the area ratio of IC and UC in the figure as 71% and 29%, respectively (Figure 1(A_cen_)). In the N_HHH_ treatment, ICs and UCs were also present in the nodule center, and the areas of IC and UC accounted for 57% and 43%, respectively. Compared with the N_LLL_ treatment, the ICs decreased and the UCs increased (Figure 1(B_cen_)). No significant differences were observed in the outer part of the nodules near the root between the two treatments (Figure 1(A_mag_,B_mag_)).

**Figure 2:**
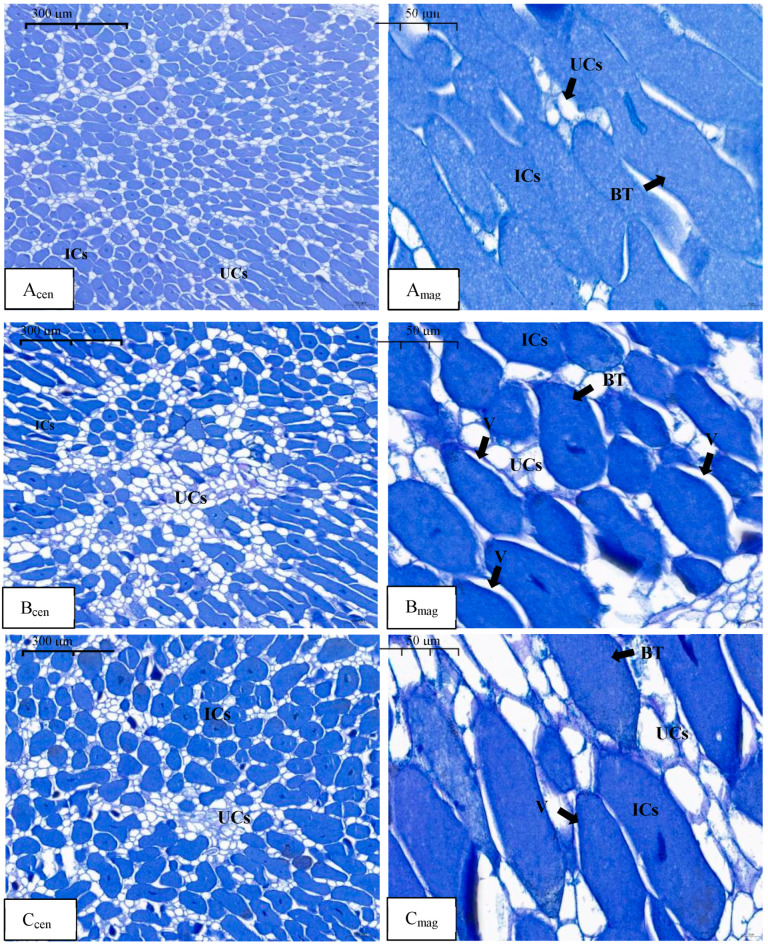
Microstructure of nodules in the N_LLL_, N_HHH_, and N_HLL_ treatments in phase II. Note: A_cen_, B_cen_, and C_cen_ are the micrographs in the nodule center of the treatments of N_LLL_, N_HHH_, and N_HLL_; A_mag_, B_mag_, and C_mag_ are the micrographs near the roots of the treatments of N_LLL_, N_HHH_, and N_HLL_; BT: rhizobium, UCs: uninfected cells, ICs: infected cells, V: vacuole; A_cen_, B_cen_, C_cen_: scale bar = 300 µm, A_mag,_ B_mag,_ C_mag_: scale bar = 50 µm.

By the end of phase II, no notable changes were observed in the N_LLL_ treatment compared to phase I (Figure 2(A_cen_,A_mag_)). The IC area ratio in Figure 2(A_cen_) is 70%. Compared with the N_LLL_ treatment, the area of UCs increased between the ICs in the nodule center in the N_HHH_ treatment, and the ICs became sparse, with an IC area ratio of 59% (Figure 2(B_cen_)). The ICs near the edge of the nodules showed irregular, predominantly spherical shapes, with an increased number of UCs between the ICs, resulting in a sparser arrangement. Vacuoles were also observed within some ICs (Figure 2(B_mag_)). In the N_HLL_ treatment, the proportion of IC area in the nodule center was 67%, and its density was close to the N_LLL_ treatment and higher than in the N_HHH_ treatment (Figure 2(C_cen_)). In the outer region of the nodules near the root, the IC density in the N_HLL_ treatment was sparse compared to the N_LLL_ treatment, but the ICs maintained a primarily rod-shaped form. Vacuoles were observed in some of the ICs (Figure 2(C_mag_)).

**Figure 3:**
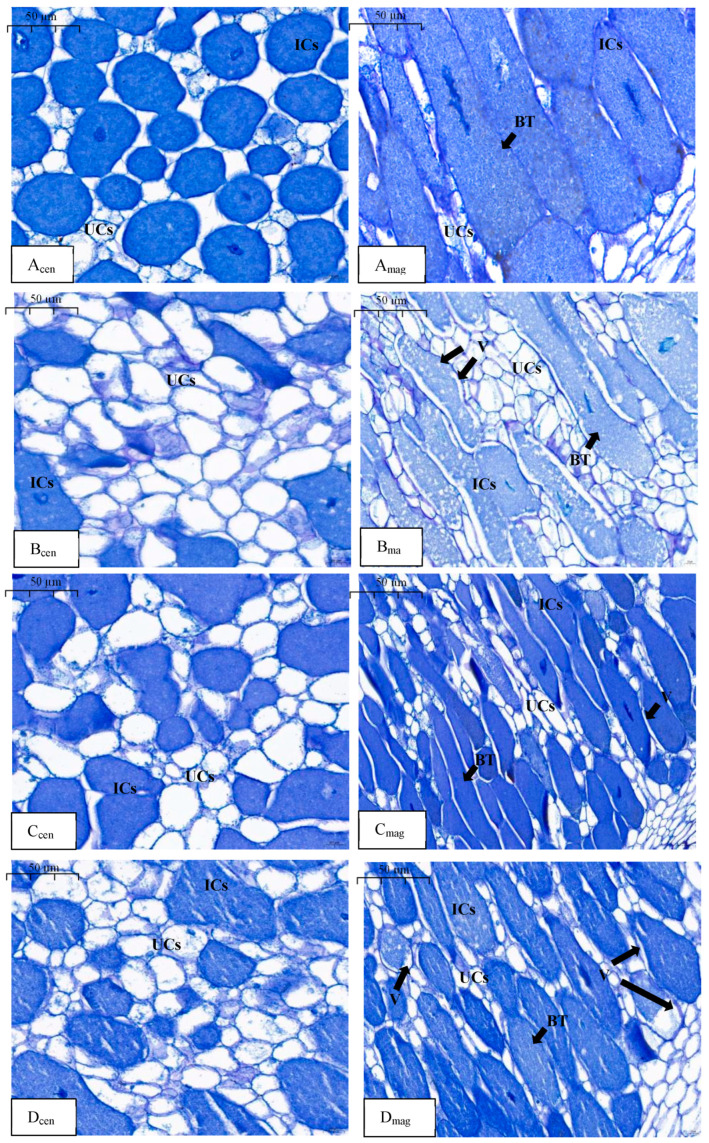
Microstructure of nodules in the N_LLL_, N_HHH_, N_HLL_, and N_HLH_ treatments in phase III. Note: A_cen_, B_cen_, C_cen_, and D_cen_ are the micrographs in the nodule center of the treatments of N_LLL_, N_HHH_, N_HLL_, and N_HLH_; A_mag_, B_mag_, C_mag_, and D_mag_ are the micrographs near the roots of the treatments of N_LLL_, N_HHH_, N_HLL_, and N_HLH_; BT: rhizobium, UCs: uninfected cells, ICs: infected cells, V: vacuole; scale bar = 50 µm.

At the end of phase III, in the N_LLL_ treatment, the ICs and UCs in the nodule center were evenly distributed, with the ICs being large and the UCs being small, the IC area ratio was 76%, and the UC area ratio was 24% (Figure 3(A_cen_)). In the N_HHH_ treatment, the nodule center was predominantly occupied by UCs, with only a few ICs scattered around. The UCs were larger in size and greater in number. The IC area ratio was 31%, and the UC area ratio was 69% (Figure 3(B_cen_)). The proportion of IC area in the nodule center of the N_HLL_ treatment was 58%, and the proportion of UC area was 42% (Figure 3(C_cen_)). The proportion of IC area in the root nodule center treated with N_HLH_ was 46%, and the proportion of UC area was 54% (Figure 3(D_cen_)). Additionally, the cell structure in the outer region of the nodules near the root showed significant differences across the four treatments. In the N_LLL_ treatment, ICs were tightly packed in a rod-like structure, filled with bacteroids (Figure 3(A_mag_)). In comparison, the N_HHH_ treatment exhibited a reduction in ICs and an increase in UCs. The bacteroids within the ICs became sparse, and numerous vacuoles formed around the bacteroids (Figure 3(B_mag_)). In the N_HLL_ and N_HLH_ treatments, the IC density was higher than in the N_HHH_ treatment but lower than in the N_LLL_ treatment, with some ICs containing small vacuoles (Figure 3(C_mag_,D_mag_)). These results indicate that the indirect supply of nitrate to soybean nodules for 3 days primarily inhibited the growth of ICs in the nodule center. As the nitrate supply continued (6–9 days), the structure and number of mature ICs near the root were also significantly affected. Following nitrate withdrawal, the number of young ICs in the nodule center increased, but the structure of mature ICs near the root did not fully recover.

## 3. Discussion

### 3.1. Effect of Nitrate on Nodulation and N Fixation of Soybean

The direct supply of nitrate to legume roots has been shown to significantly inhibit both nodule growth and nitrogenase activity [17,18,19]. Izmailov et al. (2003) noted that the nitrate assimilation activity in nodules is very low [20]. Some studies have suggested that high concentrations of exogenous nitrate have toxic effects on root nodules [21,22], limiting the oxygen supply needed for respiration in the nodules and leading to the production of toxic gases such as NO [23,24]. Additionally, nitrate can reduce the allocation of carbohydrates to the nodules [25,26]. Given these findings, some researchers have employed split-root systems to investigate further. For instance, when 14 mM nitrate was supplied for five days to one side of split-root peanut plants (*Arachis hypogaea* L.), there was little effect on the growth and N fixation capacity of the nodules on the nitrate-free side. However, when the nitrate supply was extended to 30 days, the weight, number, and nitrogenase activity of the nodules on the nitrate-free side were significantly inhibited, indicating that long-term nitrate exposure systematically regulates nodulation and nitrogenase activity in peanuts [27]. In another study, soybean roots were divided into upper and lower layers, with the lower roots supplied with 5 mM nitrate and the upper roots provided with nitrate-free nutrient solution. The results showed that the dry weight and nitrogenase activity of the upper nodules decreased compared to the control, although the number of nodules remained unchanged [28]. Using a dual-root system in soybean, nitrate was supplied to one side and it was observed that low concentrations of nitrate appeared in the nodulated side, significantly reducing nitrogenase activity [10,29,30] (Supplementary Materials). In this study, dual-root soybean plants with nodulation on one side were used as experimental materials, and nitrate was supplied, withdrawn, and resupplied on the nonnodulated side. We found that the number and weight of nodules on the nodulated side followed a pattern of increase/decrease/increase, while SNA and ARA exhibited a decrease/increase/decrease trend. These results are consistent with previous findings. To further verify these observations, we examined the microstructure of the nodules. In all three experimental phases, the control group (N_LLL_) received no nitrate, and thus showed the characteristics of mature nodules [31]. This was evident in the presence of more newly formed ICs than UCs in the central region, with only a small number of UCs scattered among the ICs (Figure 1(Acen), Figure 2(Acen) and Figure 3(Acen)). The newly formed ICs were round and gradually expanded with maturity, eventually forming a compact infected zone with densely packed bacteroids and no visible vacuoles (Figure 1(Amag), Figure 2(Amag) and Figure 3(Amag)). In the N_HHH_ treatment, where nitrate was continuously supplied to the nonnodulated side, the number of UCs in the central region increased over time, while the number of ICs decreased (Figure 1(Bcen), Figure 2(Bcen) and Figure 3(Bcen)). After nitrate was withdrawn (N_HLL_ treatment), the number of ICs in the nodule center slowly increased again (Figure 2(C_cen_) and Figure 3(C_cen_)). However, when nitrate was resupplied following withdrawal (N_HLH_ treatment), the number of ICs decreased once more (Figure 3(D_cen_)). Selker and Newcomb (1985) similarly reported a 10% increase in UCs in soybean nodules following direct nitrate application [16]. These findings suggest that nitrate supply and withdrawal significantly affect the formation of new ICs and UCs in the central region of the nodules. Moreover, with prolonged nitrate exposure on the nonnodulated side, the bacteroids within the rod-shaped ICs at the nodule periphery became sparse, and vacuoles began to form. Even after nitrate withdrawal, these altered ICs did not return to their original structure (Figure 2(C_mag_) and Figure 3(C_mag_)). Normally, vacuoles are absent in the ICs of healthy soybean nodules [32]. However, vacuoles have been observed in senescent pea (*Pisum sativum* L.) nodules, and Truchet and Coulomb (1973) suggested that vacuoles play a lytic role in nodule senescence [33]. Additionally, the presence of vacuoles may provide increased resistance to oxygen diffusion [34]. Therefore, we conclude that nitrate mainly affects the formation of new cells in the central region of the nodules, increasing the proportion of UCs over ICs in newly formed cells after nitrate application. While the formation of ICs can return to normal upon nitrate withdrawal, the structural changes in mature ICs are irreversible. Even after nitrate is removed, the rod-like structure of the infected cells cannot be restored, and the appearance of vacuoles likely reflects the onset of nodule senescence.

### 3.2. The Relationship Between Nitrate, Carbohydrate, and Nodulation N Fixation

In dark-stressed common beans (*Phaseolus vulgaris* L.), carbon supply becomes insufficient, leading to a 97% decrease in sucrose concentration within the nodules and a corresponding 95% reduction in nitrogenase activity [35]. Similarly, in bird’s-foot trefoil (*Lotus corniculatus*), the removal of the shoot caused a decline in the N fixation capacity of the nodules. Once the shoot regrew, nodule growth gradually recovered [36]. Darkness also induced the senescence of soybean nodules and impaired their N fixation ability. However, after re-exposure to light, nodule growth and N fixation recovered slowly, suggesting that the effect of carbohydrate availability on nodules is reversible [37,38]. In our previous study, we used ^13^C isotope labeling in dual-root soybeans and found that supplying nitrate to one side of the root system led to competition for carbohydrates between both sides, resulting in a reduction in ^13^C distribution to the nodules [30]. In this experiment, the concentrations of sucrose, starch, and soluble sugars in the nodules followed a pattern of decrease/increase/decrease, indicating that the indirect supply of nitrate to the nodulated side reduced carbohydrate distribution in the nodules. When nitrate was withdrawn, the distribution of carbohydrates in the nodules increased again, demonstrating that the effect of nitrate on carbohydrate allocation in the nodules is reversible.

The distribution of carbohydrates in nodules also has a significant impact on their structure. In alfalfa (*Medicago sativa* L.), insufficient photosynthate availability was found to trigger the rapid senescence of ICs [39]. Similarly, the structure of soybean nodule ICs was damaged under dark conditions but restored after 6 days of light exposure [38]. In this study, the indirect supply of nitrate to soybean nodules resulted in a reduction in carbohydrates, which was accompanied by a decrease in the number of ICs in the nodule center and an increase in UCs. Following the withdrawal of nitrate, the ICs gradually recovered and resumed growth. These findings suggest that carbohydrates in nodules play a regulatory role in the growth of both ICs and UCs. A decrease in carbohydrate distribution within nodules may lead to carbon competition between ICs and UCs. However, the exact mechanisms underlying this competition remain unclear and require further investigation.

## 4. Materials and Methods

This experiment was conducted in 2023 at the experimental base of Northeast Agricultural University, located in Xiangfang District, Harbin, Heilongjiang Province, China. The sand culture method was employed, using nodulated soybean cultivar DongDa1 (DongDa1) and nonnodulated soybean line WDD01795, L8–4858. The plant materials were obtained from Northeast Agricultural University and the Crop Research Institute of the Chinese Academy of Agricultural Sciences, respectively. Dual-root soybeans, with nodules on only one side, were prepared following the method described by Zhang et al. (2020), with two seedlings planted per pot [40]. Rhizobacteria, harvested from soybean rhizomes preserved from the previous year, were used for inoculation. These rhizomes were cleaned, crushed, and mixed with water to produce a solution containing approximately 5 g of bacteroids per liter. The supernatant was then used to irrigate the plants continuously for five days after the successful grafting of the dual-root soybean seedlings. Before the true leaves expanded (VC stage), the plants were irrigated once daily with water. From the VC to V4 stage, a nutrient solution was applied once daily, while from the V4 stage to the end of the experiment, the nutrient solution was applied twice daily (morning and evening), with each side receiving 250 mL. The nutrient solution was prepared according to the method described by Li et al. (2021), using KNO_3_ as the N source [10].

### 4.1. Experimental Design

At the VC–V4 stage, dual-root soybean systems were irrigated with a nutrient solution containing 12.5 mg·L^−1^ N. Starting from the V4 stage, both sides were irrigated with a N-free nutrient solution for 10 days. Beginning at the R1 stage (42 days after grafting), the experiment was divided into three phases: N supply, withdrawal, and resupply on the nonnodulated sides. Throughout the experiment, the N-free nutrient solution was continuously supplied to the nodulated sides. Each phase lasted for three days, with a total treatment duration of 9 days. Four treatments were designed: N_LLL_, N_HHH_, N_HLL_, and N_HLH_. The N supply treatments for the nonnodulated side were as follows: (1) N_LLL_: The N-free nutrient solution was supplied to the nonnodulated side in all three phases, serving as the control treatment; (2) N_HHH_: The nonnodulated side was supplied with 200 mg·L^−1^ N in all three phases; (3) N_HLL_: The nonnodulated side was supplied with 200 mg·L^−1^ N during phase I, followed by the N-free nutrient solution during phases II and III; (4) N_HLH_: The nonnodulated side was supplied with 200 mg·L^−1^ N during phases I and III, while the N-free nutrient solution was supplied during phase II.

### 4.2. Sampling and Measurement

Nodule nitrogenase activity: Four pots of plants were randomly selected from each treatment, and the shoot was cut along the grafting site. The nodulated lateral roots were washed with distilled water. The nitrogenase activity of the nodules was measured using the acetylene reduction method, as described by Xia et al. (2017) [41].

Sucrose concentration: Fresh samples were weighed and ground into a homogenate with 80% ethanol, followed by centrifugation at 8000× *g* for 10 min. The supernatant was quantitatively analyzed using a Waters 152 high-performance liquid chromatograph equipped with a Multospher sugar column [42].

Starch and soluble sugar concentration: Fresh samples were weighed and ground into a homogenate with 80% ethanol. The homogenate was then incubated in a water bath at 80 °C for 40 min. After cooling, the homogenate was centrifuged at 8000× *g* for 10 min at 25 °C. The supernatant was used to measure soluble sugar content, while the precipitate was used to determine starch content. The measurement was conducted using the anthrone colorimetric method as described by Bacanamwo and Harper (1996) [43].

Nodule structure: Four pots of plants were selected, and six nodules of similar size were randomly cut from the roots within 6 cm of the grafting site. These nodules were fixed in FAA solution, and their microstructure was observed following the method described by Li et al. (2023) [30].

Nitrate concentration in nodules: Fresh samples were weighed and ground into homogenate with distilled water, and extracted at 95 °C. The concentration of nitrate in the nodules was determined according to the method described by Li et al. (2021) [10].

### 4.3. Data Analysis

All statistical analyses were performed using SPSS 21.0 (SPSS Inc., Chicago, IL, USA). Before performing one-way analysis of variance (ANOVA) on the data, normality tests were performed on all data, and Duncan’s multi-range test was used. The significance level was *p* < 0.05. The area ratio of ICs and UCs in the internal structure of the nodules was calculated by Photoshop (Adobe Photoshop, 21.2.12).

## 5. Conclusions

The effect of nitrate on the new growth of ICs in the nodule center is reversible. However, for the ICs that have been damaged by nitrate supply in the early stage, the structure cannot be restored after nitrate withdrawal, which may be the main reason for the effect of indirect supply of nitrate on the N fixation ability of soybean nodules.

## Figures and Tables

**Table 1:** Number and dry weight of soybean nodules.

	Treatments	N Concentration (mg·L^−1^)	Phase I	Phase II	Phase III
Nodule Number (per plant)	N_LLL_	0-0-0	380.0 ± 14.48 a	458.8 ± 4.87 a	481.5 ± 5.89 a
	N_HHH_	200-200-200	343.3 ± 14.73 a	372.0 ± 4.88 b	370.8 ± 2.98 c
	N_HLL_	200-0-0		408.0 ± 14.20 ab	423.3 ± 2.42 b
	N_HLH_	200-0-200			382.5 ± 11.57 bc
Dry Weight (g·plant^−1^)	N_LLL_	0-0-0	0.99 ± 0.07 a	1.01 ± 0.03 a	1.11 ± 0.07 a
	N_HHH_	200-200-200	0.84 ± 0.06 a	0.85 ± 0.04 b	0.76 ± 0.05 b
	N_HLL_	200-0-0		0.90 ± 0.03 ab	1.08 ± 0.05 a
	N_HLH_	200-0-200			0.89 ± 0.07 b

Note: The values represent the mean ± standard error (n = 4), and different lowercase letters indicate that the difference between the treatments is 5% significant by Duncan analysis of variance, longitudinal comparison.

**Table 2:** Soybean nodule nitrogenase activity.

	Treatments	N Concentration (mg·L^−1^)	Phase I	Phase II	Phase III
ARA (C_2_H_4_ µmol·h^−1^·plant^−1^)	N_LLL_	0-0-0	42.71 ± 3.35 a	47.69 ± 2.24 a	42.34 ± 3.37 a
	N_HHH_	200-200-200	24.72 ± 1.73 b	25.17 ± 1.47 b	18.61 ± 2.93 b
	N_HLL_	200-0-0		29.34 ± 1.12 b	40.23 ± 2.34 a
	N_HLH_	200-0-200			21.81 ± 3.16 b
SNA (C_2_H_4_ µmol g^−1^·nodule dry mass·h^−1^)	N_LLL_	0-0-0	42.95 ± 0.60 a	47.26 ± 1.31 a	44.67 ± 4.46 a
	N_HHH_	200-200-200	30.01 ± 3.34 b	29.88 ± 1.38 b	25.49 ± 5.05 b
	N_HLL_	200-0-0		33.06 ± 2.01 b	37.19 ± 0.61 a
	N_HLH_	200-0-200			24.18 ± 1.77 b

Note: The values represent the mean ± standard error (n = 4), and different lowercase letters indicate that the difference between the treatments is 5% significant by Duncan analysis of variance, longitudinal comparison.

**Table 3:** Soybean nodule sucrose, starch, and soluble sugar concentration (mg·g^−1^).

	Treatments	N Concentration (mg·L^−1^)	Phase I	Phase II	Phase III
Sucrose	N_LLL_	0-0-0	8.90 ± 0.34 a	10.53 ± 0.21 a	10.88 ± 0.29 a
	N_HHH_	200-200-200	9.64 ± 0.03 a	9.59 ± 0.08 b	9.77 ± 0.17 b
	N_HLL_	200-0-0		9.90 ± 0.30 ab	10.81 ± 0.18 a
	N_HLH_	200-0-200			10.50 ± 0.25 ab
Starch	N_LLL_	0-0-0	7.07 ± 0.18 a	7.51 ± 0.17 a	9.94 ± 0.30 a
	N_HHH_	200-200-200	6.80 ± 0.23 a	6.94 ± 0.34 a	8.53 ± 0.67 b
	N_HLL_	200-0-0		7.39 ± 0.41 a	10.95 ± 0.80 a
	N_HLH_	200-0-200			8.97 ± 0.45 ab
Soluble sugar	N_LLL_	0-0-0	11.8 ± 0.44 a	14.47 ± 0.29 a	15.21 ± 0.24 a
	N_HHH_	200-200-200	9.98 ± 0.54 a	12.82 ± 0.26 b	12.92 ± 0.36 b
	N_HLL_	200-0-0		13.64 ± 0.60 ab	15.89 ± 0.59 a
	N_HLH_	200-0-200			12.52 ± 0.39 b

Note: The values represent the mean ± standard error (n = 4), and different lowercase letters indicate that the difference between the treatments is 5% significant by Duncan analysis of variance, longitudinal comparison.

## Data Availability

All data are included in the main text.

## Supplementary Materials

The following supporting information can be downloaded at: https://www.mdpi.com/article/10.3390/plants13243571/s1, Table S1: Nitrate concentration in soybean nodules (μg·g^−1^).

## References

[1] Sulieman S., Abdelrahman M., Tran L.-S. (2022). Carbon metabolic adjustment in soybean nodules in response to phosphate limitation: A metabolite perspective. Environ. Exp. Bot..

[2] Woodall J., Forde B. (2006). Glutamine synthetase polypeptides in the roots of 55 legume species in relation to their climatic origin and the partitioning of nitrate assimilation. Plant Cell Environ..

[3] Fujikake H., Yamazaki A., Ohtake N., Sueyoshi K., Matsubayashi S., Ito T., Mizuniwa C., Kume T., Hashimoto S., Ishioka N. (2003). Quick and reversible inhibition of soybean root nodule growth by nitrate involves a decrease in sucrose supply to nodules. J. Exp. Bot..

[4] Li S., Lyu X., Wang X., Zhao S., Ma C., Yan C., Gong Z. (2023). Assimilation of nitrate into asparagine for transport in soybeans. Agronomy-Basel.

[5] Sulieman S., Fischinger S., Gresshoff P., Schulze J. (2010). Asparagine as a major factor in the N-feedback regulation of N_2_ fixation in *Medicago truncatula*. Physiol. Plant..

[6] Matamoros M., Baird L., Escuredo K., Dalton D., Minchin F., Iturbe-Ormaetxe I., Rubio M., Moran J., Gordon A., Becana M. (1999). Stress-induced legume root nodule senescence. physiological, biochemical, and structural alterations. Plant Physiol..

[7] Wasfi M., Prioul J. (1986). A comparison of inhibition of French-bean and soybean nitrogen fixation by nitrate, 1% oxygen or direct assimilate deprivation. Physiol. Plant..

[8] Fujikake H., Yashima H., Sato T., Norikuni O., Sueyoshi K., Ohyama T. (2002). Rapid and reversible nitrate inhibition of nodule growth and N_2_ fixation activity in soybean (*Glycine max* (L.) Merr.). Soil Sci. Plant Nutr..

[9] Lyu X., Xia X., Wang C., Ma C., Dong S., Gong Z. (2019). Effects of changes in applied nitrogen concentrations on nodulation, nitrogen fixation and nitrogen accumulation during the soybean growth period. Soil Sci. Plant Nutr..

[10] Li S., Xiao F., Yang D., Lyu X., Ma C., Dong S., Yan C., Gong Z. (2021). Nitrate transport and distribution in soybean plants with dual-root systems. Front. Plant Sci..

[11] Saito A., Tanabata S., Tanabata T., Tajima S., Ueno M., Ishikawa S., Ohtake N., Sueyoshi K., Ohyama T. (2014). Effect of nitrate on nodule and root growth of soybean (*Glycine max* (L.) Merr.). Int. J. Mol. Sci..

[12] Ishikawa S., Ono Y., Ohtake N., Sueyoshi K., Tanabata S., Ohyama T. (2018). Transcriptome and metabolome analyses reveal that nitrate strongly promotes nitrogen and carbon metabolism in soybean roots, but tends to repress it in nodules. Plants.

[13] Gordon A., Skøt L., James C., Minchin F. (2002). Short-term metabolic responses of soybean root nodules to nitrate. J. Exp. Bot..

[14] Paau A., Cowles J. (1981). Bacteroid distribution in alfalfa nodules upon dark-induced senescence and subsequent partial rejuvenation. Physiol. Plant..

[15] Vauclare P., Bligny R., Gout E., De Meuron V., Widmer F. (2010). Metabolic and structural rearrangement during dark-induced autophagy in soybean (*Glycine max* L.) nodules: An electron microscopy and ^31^P and ^13^C nuclear magnetic resonance study. Planta.

[16] Selker J., Newcomb E. (1985). Spatial relationships between uninfected and infected cells in root nodules of soybean. Planta.

[17] Wang Q., Huang Y., Ren Z., Zhang X., Ren J., Su J., Zhang C., Tian J., Yu Y., Gao G. (2020). Transfer cells mediate nitrate uptake to control root nodule symbiosis. Nat. Plants.

[18] Streeter J. (1985). Nitrate inhibition of legume nodule growth and activity: II. Short term studies with high nitrate supply. Plant Physiol..

[19] Du M., Gao Z., Li X., Liao H. (2020). Excess nitrate induces nodule greening and reduces transcript and protein expression levels of soybean leghaemoglobins. Ann. Bot..

[20] Izmailov S., Bruskova R., Chechetka S., Kirnos S., Nikiforova T., Satskaya M. (2003). Nitrate assimilation function of yellow lupine root nodules?. Biol. Bull..

[21] Kanayama Y., Watanabe I., Yamamoto Y. (1990). Inhibition of nitrogen fixation in soybean plants supplied with nitrate I. Nitrite accumulation and formation of nitrosylleghemoglobin in nodules. Plant Cell Physiol..

[22] Minchin F., Becana M., Sprent J. (1989). Short-term inhibition of legume N_2_ fixation by nitrate: II. Nitrate effects on nodule oxygen diffusion. Planta.

[23] Lucinski R., Polcyn W., Ratajczak L. (2002). Nitrate reduction and nitrogen fixation in symbiotic association rhizobium-legumes. Acta Biochim. Pol..

[24] Kato K., Kanahama K., Kanayama Y. (2010). Involvement of nitric oxide in the inhibition of nitrogenase activity by nitrate in Lotus root nodules. J. Plant Physiol..

[25] Streeter J. (1981). Effect of nitrate in the rooting medium on carbohydrate composition of soybean nodules. Plant Physiol..

[26] Hacin J., Bohlool B., Singleton P. (1997). Partitioning of ^14^C-labelled photosynthate to developing nodules and roots of soybean (*Glycine max*). New Phytol..

[27] Daimon H., Yoshioka M. (2001). Responses of root nodule formation and nitrogen fixation activity to nitrate in a split-Root system in peanut (*Arachis hypogaea* L.). J. Agron. Crop Sci..

[28] Yashima H., Fujikake H., Sato T., Ohtake N., Sueyoshi K., Ohyama T. (2003). Systemic and local effects of long-term application of nitrate on nodule growth and N_2_ fixation in soybean (*Glycine max* [L.] Merr.). Soil. Sci. Plant Nutr..

[29] Lyu X., Li M., Li X., Li S., Yan C., Ma C., Gong Z. (2020). Assessing the systematic effects of the concentration of nitrogen supplied to dual-root systems of soybean plants on nodulation and nitrogen fixation. Agronomy.

[30] Li S., Wu C., Liu H., Lyu X., Xiao F., Zhao S., Ma C., Yan C., Liu Z., Li H. (2023). Systemic regulation of nodule structure and assimilated carbon distribution by nitrate in soybean. Front. Plant Sci..

[31] Newcomb W., Giles K., Atherly A. (1981). Nodule morphogenesis and differentiation. Biology of the Rhizobiaceae.

[32] Gordon A., Thomas B., Reynolds P. (1992). Localization of sucrose synthase in soybean root nodules. New Phytol..

[33] Truchet G., Coulomb P. (1973). Mise en évidence et évolution du système Phytolysosomal dans les cellules des différentes zones de nodules radiculaires de Pois (*Pisum sativum* L.). Notion d’hétérophagie. J. Ultrastruct..

[34] Sujkowska M., Górska-Czekaj M., Bederska M., Borucki W. (2011). Vacuolar organization in the nodule parenchyma is important for the functioning of pea root nodules. Symbiosis.

[35] Gogorcena Y., Gordon A., Escuredo P., Minchin F., Witty J., Moran J., Becana M. (1997). N_2_ fixation, carbon metabolism, and oxidative damage in nodules of dark-stressed common bean plants. Plant Physiol..

[36] Vance C., Johnson L., Miller S., Groat R. (1982). Birdsfoot trefoil (*Lotus corniculatus*) root nodules: Morphogenesis and the effect of forage harvest on structure and function. Can. J. Bot..

[37] Pfeiffer N., Malik N., Wagner F. (1983). Reversible dark-induced senescence of soybean root nodules. Plant Physiol..

[38] Cohen H., Sarath G., Lee K., Wagner F. (1986). Soybean root nodule ultrastructure during dark-induced stress and recovery. Protoplasma.

[39] Vance C., Johnson L., Halvorsen A., Heichel G., Barnes D. (1980). Histological and ultrastructural observations of *Medicago sativa* root nodule senescence after foliage removal. Can. J. Bot..

[40] Zhang R., Wang C., Teng W., Wang J., Lyu X., Dong S., Kang S., Gong Z., Ma C. (2020). Accumulation and distribution of fertilizer nitrogen and nodule-fixed nitrogen in soybeans with dual root systems. Agronomy.

[41] Xia X., Ma C., Dong S., Xu Y., Gong Z. (2017). Effects of nitrogen concentrations on nodulation and nitrogenase activity in dual root systems of soybean plants. Soil Sci. Plant Nutr..

[42] Tiwari M., Mhatre S., Vyas T., Bapna A., Raghavan G. (2023). A validated HPLC-RID method for quantification and optimization of total sugars: Fructose, glucose, sucrose, and lactose in eggless mayonnaise. Separations.

[43] Bacanamwo M., Harper J. (1996). Regulation of nitrogenase activity in *Bradyrhizobium japonicum*/soybean symbiosis by plant N status as determined by shoot C:N ratio. Physiol. Plant..

