# Supplementary material for: A Study on the Effect of Indirect Nitrate Supply on the Nitrogen Fixation Capacity of Soybean Nodules
Source: Plants (Basel). 2024 Dec 21;13(24):3571. doi: 10.3390/plants13243571 (PMC11678621; doi:10.3390/plants13243571)
Supplement: Supplementary file 1 [file plants-13-03571-s001.zip › plants-3340488-supplementary.pdf]

Table S1. Nitrate concentration in soybean nodules ($\mu g \cdot g^{-1}$)

Treatments	N Concentration ($mg \cdot L^{-1}$)	Phase I	Phase II	Phase III
N_{LLL}	0-0-0	48.4±3.74 b	58.3±1.13 c	84.7±4.21 c
N_{HHH}	200-200-200	138.0±7.85 a	196.5±1.11 a	238.1±26.4 a
N_{HLL}	200-0-0		130.7±9.94 b	99.4±4.48 c
N_{HLH}	200-0-200			165.7±16.04 b

Note: The values represent the mean ± standard error (n=4), and different lowercase letters indicate that the difference between the treatments is 5% significant by Duncan analysis of variance, longitudinal comparison.
